# Inaccuracy of Death Certificate Diagnosis of Tuberculosis and Potential Underdiagnosis of TB in a Region of High HIV Prevalence

**DOI:** 10.1155/2012/937013

**Published:** 2012-03-19

**Authors:** Theresa T. Liu, Douglas Wilson, Halima Dawood, D. William Cameron, Gonzalo G. Alvarez

**Affiliations:** ^1^Division of Infectious Diseases, The Ottawa Hospital, University of Ottawa, Ottawa, ON, Canada K1H 8L6; ^2^Department of Medicine, Edendale and Grey's Hospitals, Pietermaritzburg, KwaZulu-Natal 3200, South Africa; ^3^Division of Respirology, The Ottawa Hospital, University of Ottawa, Ottawa, ON, Canada K1H 8L6

## Abstract

Despite the South African antiretroviral therapy rollout, which should reduce the incidence of HIV-associated tuberculosis (TB), the number of TB-attributable deaths in KwaZuluNatal (KZN) remains high. TB is often diagnosed clinically, without microbiologic confirmation, leading to inaccurate estimates of TB-attributed deaths. This may contribute to avoidable deaths, and impact population-based TB mortality estimates. *Objectives*. (1) To measure the number of cases with microbiologically confirmed TB in a retrospective cohort of deceased inpatients with TB-attributed hospital deaths. (2) To estimate the rates of multi-drug resistant (MDR) and extensively drug resistant (XDR) TB in this cohort. *Results*. Of 2752 deaths at EDH between September 2006 and March 2007, 403 (15%) were attributed to TB on the death certificate. 176 of the TB-attributed deaths (44%) had a specimen sent for smear or culture; only 64 (36%) had a TB diagnosis confirmed by either test. Of the 39 culture-confirmed cases, 27/39 (69%) had fully susceptible TB and 27/39 (69%) had smear-negative culture-positive TB (SNTB). Two patients had drug monoresistance, three patients had MDR-TB, and one had XDR-TB. *Conclusions*. Most TB-attributed deaths in this cohort were not microbiologically confirmed. Of confirmed cases, most were smear-negative, culture positive and were susceptible to all first line drugs.

## 1. Introduction

Tuberculosis (TB) is a global problem, with approximately 9 million new cases per year; of these cases, 1.1 million cases per year are due to coinfection with TB and human immunodeficiency virus (HIV) [1]. Approximately 82% of the global cases of HIV-associated TB are in the African Region, and South Africa accounts for a significant proportion of cases in the region [1]. In 2010, South Africa had the third largest absolute number of TB cases in the world, with an incidence of 0.4–0.59 million [1], or 998 cases/100,000 population [2]. 73% of new TB cases in South Africa in 2007 were HIV positive [2]. Tuberculosis is the leading cause of death in South Africa by clinically determined death notification forms [3]. Edendale Hospital (EDH) is an 860-bed district and regional hospital in Pietermaritzburg, KwaZuluNatal (KZN), South Africa, and is the regional TB referral centre. It serves a mainly Black African population of approximately 1.6 million [4]. HIV prevalence rates in regional antenatal clinics vary between 40 and 50% [5], and the rate of TB disease in KZN is estimated as between 773–1008/100,000 [3]. The high TB mortality rate in this region is likely associated with the high HIV prevalence and/or the presence of drug-resistant TB known to be in the region. Recently, an outbreak of XDR-TB (extensively drug-resistant TB) occurred in a rural hospital approximately 100 km from EDH [6]. The median survival time in this outbreak was two weeks after obtaining sputum samples. Almost all of these patients were HIV positive [6]. Information on the patients who died indirectly or directly as a result of TB at EDH may reveal high proportions of specific patterns of drug resistance, specifically MDR-TB (multidrug-resistant TB) and XDR-TB, which are associated with high mortality. A recent postmortem study at EDH revealed that the accuracy of limited autopsy diagnosis of TB is approximately 47%; furthermore, 42% of patients who died who previously were not suspected to have TB were actually TB culture positive on limited autopsy [7]. The present study set out to measure the number of patients with a TB diagnosis by sputum smear and culture in TB-attributed hospital deaths and to estimate the rates of MDR- and XDR-TB in TB-attributed hospital deaths.

## 2. Methods

A retrospective cohort study of all adult inpatients admitted to the EDH wards that died with a diagnosis of TB during a seven-month period between September 2006 and March 2007 was undertaken. A list from the EDH mortuary of patients who died during the study period with a diagnosis of TB recorded on their death certificate was recorded and cross-referenced with the EDH laboratory AFB (acid fast bacilli) smear registry and culture and drug susceptibility registry. The study period was chosen due to the availability of data from the hospital mortuary and mycobacteriology laboratories. Physicians filling out the death certificates could include up to three diagnoses. Ethics approval for the audit was obtained from the Edendale Hospital Ethics Committee. One of the authors (T.I.Liu) collected and cross-referenced the data manually. The mortuary lists of TB-attributed deaths and the AFB smear registry were on paper. The TB culture and susceptibility data was computerized, and was accessed remotely through the mycobacteriology reference laboratory at Inkosi Albert Luthuli Central Hospital.

## 3. Inclusion Criteria

All adult inpatients (>18 years of age) at EDH were eligible for inclusion in the study if they died in hospital during the study period, and if TB was documented by a hospital physician on their death certificate as being a direct or indirect cause of death. Death certificate data was only available from the mortuary for patients who died on an adult inpatient ward at EDH.

## 4. Definitions

AFB smear results were recorded as negative, scanty, 1+, 2+, or 3+, as defined by the South African National TB Control Programme Practical Guidelines [8]. Sputum samples are not routinely sent for culture in this resource limited setting, unless the patient has been on TB treatment in the past, is persistently smear-positive on treatment, or is persistently symptomatic despite two previous negative smears and a course of broad-spectrum antibiotics, or if recurrent/relapsed TB disease is suspected [8]. Smear and culture methodology are in accordance with international guidelines using florescent microscopy, liquid media for initial culture, and Middlebrook 7H10 solid media for drug susceptibility testing. Positive TB cultures that were sent for susceptibility testing were classified as pansensitive to all first line drugs, monoresistant to a single agent, multidrug resistant (MDR), or extensively drug resistant (XDR). MDR-TB is defined as resistance to at least isoniazid (INH) and rifampin (RIF). XDR-TB is defined as resistance to INH and RIF as well as resistance to the fluoroquinolones and any one of the second-line injectable antituberculous drugs (amikacin, kanamycin, or capreomycin), as per the October 2006 revised definition by the World Health Organization (WHO) Global XDR-TB Task Force [9].

## 5. Results

### 5.1. Demographics

The average age at death was 36 years (standard deviation 14). Approximately one-third of subjects were male, one-third was female, and for the remaining one-third, gender was unavailable (Table 1).

### 5.2. In Hospital TB Deaths

Of 2752 inpatient deaths in the seven-month study period from September 2006 to March 2007, 403 deaths (14.6%) were attributed to TB according to the death certificate. An average of 58 TB deaths per month occurred during the study period.

### 5.3. Site of Disease (Tables 1 and 2)

Pulmonary TB (PTB) was the most common site of infection, with 227/403 (56%) cases (Tables 1 and 2). 118 (29%) had extrapulmonary TB (EPTB). Meningitis, miliary/disseminated, and peritoneal TB were the most common types of EPTB. 58 subjects (14%) did not have a specific site of TB infection documented on the death certificate and did not have any supportive AFB smear or culture results to help identify disease site.

### 5.4. TB Culture and Susceptibility

**Figure 1:**
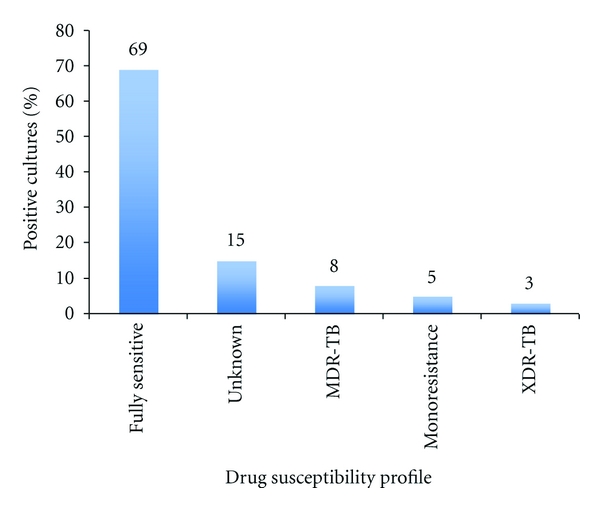
Drug susceptibility profile of positive cultures.

176/403 (44%) of the patients had a result registered for AFB smear or culture, and of those, only about 64/176 (36%) had a TB diagnosis confirmed with either test (Table 2). Of the 39 culture-confirmed cases, 27 (69%) were smear negative, of which 12 were sputum samples, 7 lymph node or abscess aspirates, 6 pleural or peritoneal fluid samples, and 1 CSF sample. Of the 39 culture-confirmed cases, 27/39 (69%) had fully susceptible TB (Figure 1). Two patients had drug monoresistance, three patients had MDR-TB, and one had XDR-TB. Six patients (15.4%) had culture-positive TB with no susceptibility results available. Of the six cases of drug-resistant TB of any kind, only two had been previously designated as MDR-TB suspects (one had culture-confirmed MDR-TB, and the other had rifampin monoresistance). Of the fifteen MDR-TB suspects, only one patient had confirmed MDR-TB. The one case of XDR-TB was not previously suspected to have had drug-resistant TB. In two patients, *Mycobacteria* other than TB (MOTT) was isolated (one grew MOTT alone, and another grew both TB and MOTT).

## 6. Discussion

The present study demonstrates that TB mortality remains high in this region, and that the majority of patients that died with a diagnosis of TB did not have a microbiologically confirmed diagnosis by AFB smear or culture. Of those with a confirmed diagnosis, the majority were smear negative, culture positive, and susceptible to all first line drugs; only one case of XDR-TB was identified. The findings likely reflect the known high prevalence of HIV in the region, which has increased the SNTB rates resulting in diagnostic delays in the community. Many HIV-positive patients have AFB smear-negative TB disease, resulting in diagnostic delays, and worsening of their clinical condition to the point that they require hospital admission. Following admission, confirmation of TB is further delayed by the inability to obtain an adequate diagnostic specimen from moribund patients, the patients' inability to produce sputum, and the lack of availability of appropriate invasive diagnostics. Mycobacteria cultures may not be sent on inpatients already established on antituberculosis therapy at a primary care clinic and admitted with a superimposed condition (such as community acquired pneumonia). Once a specimen is obtained, the time to confirmation of TB is further delayed by the time required for laboratory culture and sensitivity testing. All of these reasons contribute to low rates of microbiologic confirmation of a clinical TB diagnosis in this setting.

In our cohort of 39 culture-confirmed cases among all TB-attributed deaths, 69% had isolates susceptible to all first-line drugs. Despite having effective antituberculosis medications, many people in this region are dying of pansusceptible TB, most likely due to the difficulties encountered in the diagnosis of TB in HIV-infected patients. HIV-infected patients are less likely to have AFB smear-positive disease or cavitary TB and are more likely to have disseminated or EPTB [7]. In this study, the proportion of PTB (56%) to EPTB (29%) cases was approximately 2 : 1. In a previous study at EDH in 2001, a similar ratio of PTB to EPTB cases (65% compared to 36%) was noted [4]. The expected ratio of PTB to EPTB cases ranges from 5 : 1 [10] to 9 : 1 [1]; according to the WHO 2011 Global Tuberculosis Control Report, in high burden countries in 2010, the distribution of PTB : EPTB cases was approximately 9 : 1 [1]. The disproportionately high number of EPTB cases at EDH is likely due to high rates of undiagnosed HIV/TB coinfection in admitted patients. Out of fifteen MDR suspects in our cohort, only one had confirmed MDR-TB. This finding underscores the fact that MDR-TB and XDR-TB are difficult to distinguish clinically from pansusceptible TB disease, and therefore routine airborne infection control practices should be undertaken in all admitted TB suspects, regardless of the suspicion of drug resistance. However, given the TB burden and limited resources in this part of the world, the logistics of infection control remains a significant challenge. 

A recent postmortem study by Cohen et al. that was done at EDH one year after the present study was completed showed that in a representative sample of 240 inpatient deaths between October 2008 and August 2009, 110 (47%) were culture positive on limited autopsy pooled sampling of respiratory tract secretions and core biopsies of lung, liver, and spleen [7]. 121 of these inpatient deaths (50.4%) were not on TB treatment at the time of death, and 119 (49.5%) of them were on TB treatment [7]. Of those 119 cases on TB treatment, 64 (58%) were culture positive, and 55 (46%) were culture negative [7]. Therefore 55/240 (23%) of these cases would have been attributed to TB in hospital deaths based on the death certificate, in the absence of a positive culture result. In this study, which was done at the same center as our study only one year later, the TB diagnosis rate (by culture-positive limited autopsy samples) among TB suspects was 58%, which demonstrates the significant underestimation of TB hospital attributed deaths in this region when using only the death certificate. The death certificate diagnosis rate of TB at EDH was less accurate than limited autopsy likely due to the challenges in obtaining sputum or other tissue as outlined previously, as well as the high rates of smear-negative TB associated with high HIV prevalence.

Most patients with culture-confirmed TB had drug-susceptible TB, which was perhaps surprising giving the documented prevalence of MDR- and XDR-TB in this region of South Africa [1]. A selection bias that may have affected our results is that admitted patients who died of TB may have died immediately following admission, prior to appropriate samples being sent for testing. Information on length of hospital stay prior to death was not available for our subjects. A selection bias could have resulted from these patients, since they may have had a higher rate of resistance; it is known that patients infected with XDR-TB have a shortened survival [6]. A recent study in KZN demonstrated markedly high early mortality rates for HIV/drug-resistant TB coinfection; 40% of HIV/MDR-TB and 51% of HIV/XDR-TB cases died within 30 days of sputum collection [11]. Because patients in our study may have died rapidly after admission, they may have been too debilitated to give an adequate sputum sample for culture or undergo invasive testing. The selection bias would apply to the 226 subjects that died without having a specimen sent for AFB smear or culture; however these patients would have had the same random chance of having either a sputum sample or another type of specimen sent to the laboratory prior to admission. We would have expected that patients that were at higher risk of resistance would have had sputum sent for testing, as per the South African National TB Control Program guidelines [8]. Furthermore, patients infected with MDR- or XDR-TB may have died before they even presented to hospital and thus would not be accounted for in hospital mortality registries. From the WHO 2011 Global Tuberculosis Control Report, an estimated 1.8% of incident TB cases in South Africa were MDR-TB [1]. In our study, from the 39 culture-confirmed TB deaths, we found 3 cases of MDR-TB and 1 case of XDR-TB. Although we do not know the South African MDR-TB mortality rate, this is in keeping with the published rates of incident MDR-TB cases in South Africa [1].

Another limitation of our study is that we were not able to determine the HIV status of the majority of our subjects since at the time of the study point of care testing was not available. It is well documented that HIV/TB coinfection increases the rapidity of clinical deterioration and increases the risk of death [12–15]. Stigma associated with HIV/AIDS may influence people's willingness to be tested for HIV—“retroviral disease” was a common euphemism used on the death certificates to avoid labeling patients as HIV positive. Of the four patients with either MDR-TB or XDR-TB in our study, three had documented HIV infection. EDH has since developed a program in which a designated team of lay health workers offer point of care HIV testing and assist in obtaining sputum samples to all admitted TB suspect patients [16]. Future studies should focus on streamlining which patients should get smears and cultures sent to the laboratory, given the high number of specimens processed and the limited resources of regional laboratories.

Misclassification bias may have occurred in physician-completed death certificates. All over the world, physicians often have to make a judgment call on the ultimate cause of death of the deceased. Determining the cause of death in patients with active TB disease is a challenge and thus may have resulted in misclassification. A review of 60 cases of pulmonary TB in a hospital in Manchester, Great Britain, in 1983, demonstrated that the actual cause of death in admitted inpatients with TB can be difficult to ascertain [17]. In this study, the pulmonary TB in-hospital mortality rate over a 7-year period (1974–1981) was 7%; of the 60-patients that died, 36 died while on treatment for TB. 10 patients died with unequivocal evidence of overwhelming tuberculosis infection, 6 died for unrelated reasons, and 4 died of drug-related conditions. The remaining 16 (27%) died suddenly or unexpectedly for unclear reasons—13 of these patients underwent autopsy, and 9 patients still did not have an identifiable cause of death on postmortem [17]. Despite autopsy, there were still difficulties in identifying the actual cause of death in patients with pulmonary TB. Verbal autopsies, in which fieldworkers interview close family members to complete a questionnaire asking about a patient's symptoms and course of illness prior to their death, have been validated as a useful tool in South Africa [18, 19]. In a study looking at mortality in South African gold miners with pulmonary TB, the cause of death was determined by reviewing clinical records and from the results of limited autopsies of the heart and lungs [14]. In this population, limited autopsies are mandated for purposes of compensation but have also provided further information on patterns of TB disease and drug susceptibility in South Africa.

## 7. Conclusions

Our study demonstrates that hospitalized TB patients continue to experience a high mortality, despite major efforts to roll out antiretrovirals (ARVs) in this high HIV prevalence region, and that the majority of patients that died with TB identified as the cause of death on the death certificate did not have a TB diagnosis confirmed by AFB smear or culture. The majority of the TB suspects that were tested for and died of TB had a fully susceptible organism. The cohort studied detected only one XDR-TB case, despite significant XDR-TB outbreaks associated with high mortality in a nearby hospital. Death certificate diagnosis of TB was less accurate than limited autopsy diagnosis of TB at the same hospital, during a similar time period.

## 8. Lessons Learned

This study emphasizes the importance of active TB and HIV case finding among inpatients at EDH, such as through the work of EDH's “TB Warriors” program in coordinating laboratory testing treatment and follow-up of TB suspects [16]. In this area of high HIV endemicity, routine testing for HIV should continue to be offered to TB patients to help identify more HIV/TB coinfected people, who are at higher risk of dying. Diagnosing TB quickly and efficiently in this setting continues to be a challenge given the high prevalence of both TB and HIV, but it is limited by hospital and laboratory resources. There is also an urgent need for diagnostic tools that will rapidly diagnose TB in patients too ill to produce sputum. Clinicians should have a low threshold for sending sputum for TB culture in HIV positive patients, given the greater possibility of smear negative and/or drug-resistant TB. The earlier patients are diagnosed, the greater chance of starting both TB and HIV treatment in a timely fashion, thus reducing HIV/TB-associated mortality.

## Figures and Tables

**Table 1:** Clinical and demographic characteristics.

Total number of TB deaths	*n* = 403
Sex, female (%)	144 (36%)
Sex, male (%)	122 (30%)
Sex not documented on death certificate	137 (34%)
Average age at death, years (standard deviation)	36 (14)
Pulmonary TB (%)	227 (56%)
EPTB (%)	118 (29%)
Miliary/disseminated	30 (7%)
Meningitis	59 (15%)
Peritonitis	20 (5%)
Spine	5 (1.2%)
Lymphadenitis	3 (0.7%)
Pericardium	1 (0.2%)
TB source unknown	58 (14%)
Documented HIV positive*	22 (5.4%)
Presumed HIV positive**	55 (13.6%)
HIV status unknown	326 (81%)

*Documented HIV positive via CD4 or HIV viral load records; **Presumed HIV positive due to death certificate diagnosis of HIV, retroviral disease (RVD), or AIDS-defining opportunistic infection.

**Table 2:** Number of TB-attributed inpatient deaths, by disease site and diagnostic test.

Diagnosis	Number (% of total deaths)	AFB smear done	AFB smear positive	Culture done	Culture positive	Smear or culture positive	Totals (% of total deaths)
PTB	227 (56)	61	28	59	29	47	227 (56)
EPTB							
Disseminated/miliary	30 (7.4)	11	1	9	4	5	
TB meningitis	59 (14.6)	10	0	13	2	2	
TB abdomen	20 (4.9)	3	1	7	1	1	118 (29)
TB spine	5 (1.2)	1	0	1	0	0	
TB pericardium	1 (0.2)	0	0	1	0	0	
TB source unknown	58 (14)	8	1	9	2	3	58 (14)
MOTT (*Mycobacteria *other than TB)	2 (0.5)	0	0	2	2	2	2 (0.5)
